# Melanoma Disparities in Hispanic Populations: Socioeconomic Challenges and Cultural Barriers

**DOI:** 10.1155/jskc/3133773

**Published:** 2025-09-23

**Authors:** Tarek Zieneldien, Sophia Ma, Janice Kim, Bernard A. Cohen

**Affiliations:** ^1^Johns Hopkins University School of Medicine, Baltimore, Maryland, USA; ^2^Michigan State University College of Osteopathic Medicine, East Lansing, Michigan, USA; ^3^Department of Dermatology, The Johns Hopkins Hospital, Baltimore, Maryland, USA

## Abstract

Hispanic and Latino melanoma patients face significant disparities in patient outcomes and survival rates due to challenges in melanoma care, including later-stage diagnoses, disproportionate financial burdens, and cultural differences. Healthcare insurance status is an important contributing factor to these disparities, with uninsured Hispanic patients being more likely to delay seeking medical attention and face higher out-of-pocket costs. Socioeconomic factors, including lower income levels, limited education, and occupations that may increase UV exposure exacerbate these disparities. Additional factors such as inadequate patient education, language barriers, and inadequate health education campaigns also limit melanoma awareness, prevention, and healthcare access in Hispanic populations. Consequently, there is an urgent need for culturally tailored interventions, such as community-based, targeted health campaigns and enhanced, culturally sensitive provider training to better address these disparities and improve melanoma outcomes in Hispanic and Latino populations.

## 1. Introduction

Melanoma is the deadliest form of skin cancer as, arising from the malignant transformation of melanocytes within the basal layer of the epidermis, it is prone to metastasizing [1]. Melanocytes are responsible for producing melanin, which absorbs potentially harmful ultraviolet (UV) radiation and visible light [2]. While early stages of melanoma can be surgically removed, melanoma spreads locally, regionally, and distally [3]. The risk of metastasis is dependent on the depth of invasion and ulceration of the primary lesion [3]. Melanoma is most commonly diagnosed in non-Hispanic White (NHW) populations, particularly individuals with fair skin tones who have had extensive sun exposure [4]. Although the overall incidence of melanoma is lower among Hispanic populations, they experience significant disparities in outcomes as they are more likely to be diagnosed at later, more aggressive stages, resulting in worse outcomes compared to NHWs [5]. This disparity is attributed to a multitude of factors, including limited access to healthcare, lower awareness of melanoma risks, and cultural misinformation about melanoma. These challenges highlight a population that is relatively understudied in skin cancer research, as well as the importance of increased awareness in mitigating ethnic disparities in melanoma-related mortalities.

In the United States, the Hispanic population, including both Latinos and Hispanics, are the second-largest racial or ethnic group, accounting for 19.1% of the total population [6]. By 2060, Hispanics are predicted to compose one-third of the United States population. Despite a lower melanoma incidence among Hispanics, those affected are more likely to present with stage III disease and an increased risk of more distant metastasis, resulting in poorer survival rates [7]. A study conducted on melanoma cases in Miami-Dade County found that a higher percentage of Hispanic patients presented with late-stage diagnoses, including distant-stage melanoma, compared with NHWs [8]. Additionally, another study conducted using the National Cancer Database found that Hispanic patients were more likely to present with stage III/IV melanoma, greater overall mortality, and higher rates of acral lentiginous melanoma than NHW patients [9]. These studies demonstrate the higher rates of late-stage diagnosis in Hispanic populations and the ensuing survival rate disparities.

Many factors contribute to the later-stage diagnoses observed in Hispanic populations by hindering timely treatment, including restricted healthcare access, socioeconomic determinants, insufficient patient education about melanoma, language barriers, and cultural practices or beliefs. The lack of access to health insurance for Hispanic communities results in individuals delaying their visits to healthcare professionals in a timely manner, and the out-of-pocket costs can hinder their ability to seek both diagnosis and treatment [10]. Additionally, the lack of health insurance limits preventive care, where patients may not receive routine skin checks or preventative screenings that could detect melanoma in its early stages and prevent late-stage diagnosis [11]. Moreover, Hispanic patients may only seek healthcare professionals when symptoms become severe, which results in them being diagnosed after melanoma has progressed to later stages [12]. Together, these elements contribute to a cycle of underdiagnosis, delayed treatment, and dually poor outcomes in terms of both health and financial burden, underscoring the urgent need to address these disparities to improve healthcare coverage and achieve better health outcomes.

To provide clarity and direction in addressing these complex challenges, this narrative review was conducted by identifying and evaluating relevant articles from PubMed, Google Scholar, and other academic databases (2000–2025) using search terms such as “melanoma” or “cutaneous melanoma” combined with terms related to inequities in the examined population including “disparities,” “Hispanic,” “Latino,” “healthcare access,” “socioeconomic determinants,” “insurance,” “cultural competence,” “health education,” “language barriers,” “acculturation,” “public health campaigns,” “prevention,” “intervention,” “artificial intelligence,” and “medical education.” Studies were included if they addressed clinical characteristics, epidemiology in Hispanics, healthcare access or insurance, socioeconomic or occupational determinants, prevention or education, language or cultural barriers, technological advances, or medical education initiatives. Non-English articles, case reports, and studies unrelated to melanoma disparities in Hispanics were excluded. Over 70 studies, covering epidemiological data, systematic reviews, qualitative research, expert commentaries, and intervention trials, were assessed for relevance and contribution to current knowledge and guiding future directions in the field. In summary, melanoma disparities in Hispanic populations are examined through a synthesis of current evidence, with particular emphasis on the following areas (Table 1):

• Examines the epidemiology of melanoma among Hispanics, highlighting their higher likelihood of late-stage diagnosis and worse survival compared to NHWs.
• Analyzes structural and socioeconomic barriers such as low insurance coverage, immigration-related access challenges, and occupational risk factors prevalent in Hispanic communities.
• Explores cultural and linguistic factors, including health literacy, acculturation, and the role of familism, that influence prevention behaviors and healthcare utilization.
• Reviews existing targeted public health campaigns, including digital and community-based educational strategies tailored to Hispanic populations.
• Highlights the need for enhanced provider training and a more diverse healthcare workforce to improve communication and trust.
• Proposes future priorities for research and policy to reduce melanoma disparities and promote equitable care in this rapidly growing segment of the U.S. population.

In this review, we begin by recognizing healthcare insurance challenges affecting melanoma outcomes in Hispanic populations, followed by an examination of the role of socioeconomic determinants. We then discuss disparities related to limited education and inadequate health education campaigns. Cultural and linguistic factors, including language barriers and acculturation, are subsequently explored. The final sections address current prevention efforts and future directions, including early detection strategies, public health campaigns, medical education initiatives, provider training, and proposed areas for future investigation.

## 2. Healthcare Insurance

There is a low rate of health insurance, particularly among noncitizen Latino immigrants, of whom less than half report health insurance coverage due to various structural and sociopolitical barriers that limit access to healthcare [13]. While the Affordable Care Act Medicaid expansion led to a decreased proportion of preventable hospitalizations for Hispanic populations, uninsurance rates are still relatively high compared with NHW and Black populations [17, 46]. This may, in part, be due to the discretion on expanding Medicaid being state specific; Florida and Texas, two states with large Latinx populations, have not participated in the expansion [17]. Another contributing factor to Hispanic individuals' hesitancy to enroll in Medicaid includes fears about deportation associated with undocumented and legal residency status concerns [17]. The status of not having a green card was estimated to have caused 107,956–192,905 Latino immigrants in California alone to decide not to enroll in Medicaid during the 2017–2020 period [47]. On a local level, Hispanic patients may receive healthcare coverage through private–public partnerships, student-run clinics at academic centers, or mobile clinics [14]. This expands access to care but differs by resource, location, and eligibility. Despite this, 48% of Hispanic immigrants who have been living in the United States for 10 years or less state that they have a primary care provider. In addition, a cross-sectional study conducted by Zagona-Prizio et al. found that, compared with NHWs, Hispanic patients visit primary care providers more frequently than outpatient dermatologic clinics for skin conditions [15]. These patients may also reside in underserved areas where specialty care, such as dermatology or cancer specialists, may not be available, further exacerbating the delays in diagnosis and treatment [16]. Thus, addressing disparities in access to dermatologic care is critical to improving outcomes for melanoma in Hispanic populations.

### 2.1. Socioeconomic Determinants

Poverty, economic opportunity, and level of educational attainment are community factors that have also been demonstrated to influence health and outcomes. Currently, limited research has been conducted on the community factors influencing melanoma outcomes in Hispanics, with existing studies relying on composite measure data derived from population-based cancer registries. This makes it more challenging to decipher which determinants result in melanoma inequities among Hispanic subgroups. Among Hispanics, high-SES individuals make up one-third of those with melanoma, while more than 60% of NHWs with melanoma incidence were high-SES individuals [48]. This opposing SES gradient between Hispanics and NHWs may suggest a unique set of exposure risks in the Hispanic population.

Furthermore, a large number of Hispanics work in outdoor occupations, such as transportation, construction, landscaping, agriculture, and maintenance [18]. These occupations are associated with significant chemical exposure and UV radiation. A population-based sample of outdoor workers conducted by Day et al. found that 69% of the sample reported rarely or never wearing sunscreen while working, a risk factor for melanoma. Likewise, in a cross-sectional study of Latino Migrant Farmworkers in North Carolina, it was found that 90.8% of the workers rarely used sunscreen and had low levels and nearly 80% had low perception and level of knowledge regarding skin cancer risks [19, 20].

Subsequently, the different subgroups of Hispanics in the United States have different migration and settlement patterns. Nearly all Puerto Ricans are native-born US citizens, and more than two-thirds of Mexican Americans in the United States are US-born. On the other hand, most other national origin groups are principally foreign-born [49]. To illustrate some of the differences in healthcare habits of Hispanic immigrants, 55% of those who have been living in the United States for 10 years or less have stated that they have seen a healthcare provider in the past year, compared with 63% for those who have been living in the United States for 11–20 years and 77% for those who have been living in the United States for longer than 20 years [50]. This heterogeneity in Hispanic subgroups is also a potential opportunity for exploring and understanding how cultural, environmental, genomic, and socioeconomic factors contribute to melanoma development.

### 2.2. Limited Education

Education level is a crucial link between socioeconomic status and health, potentially predicting cancer outcomes [21]. It has been estimated that if the cancer death rates of the American population equaled that of college-educated Americans, approximately 22% of US cancer deaths could be avoided [22]. Even though a direct biological connection does not exist between the education level and cancer risk, it is believed that a higher education level promotes healthier habits and more effective self-management. Cokkinides et al. examined trends in melanoma mortality among NHWs and found that melanoma mortality declined significantly between 1993–1997 and 2003–2007 in men and women [23]. However, this trend was only applicable to individuals with high educational attainment (13 years or more of education). Interestingly, nonsignificant increases in mortality were found among the least-educated individuals—particularly in men [23]. Studies have shown that levels of college graduation for NHWs are notably higher when compared with Latinos and that only 11% of foreign-born Latinos have a Bachelor's degree compared with 18% of those that are native-born [21, 24]. Thus, a lack of education leads to widening disparities in melanoma outcomes, emphasizing the need for early detection strategies.

### 2.3. Inadequate Health Education Campaigns

Health education and communication campaigns play pivotal roles in shaping risk behaviors and health perceptions, particularly those associated with skin cancer and melanoma. Studies show that mass media campaigns can influence risky behaviors at the population health level [51]. Media advocacy and public relations campaigns are promising complementary strategies to conventional mass media campaigns. In the past, mass media campaigns have been effective at increasing screening for breast, cervical, and colorectal cancer [52]. Nonetheless, even though public messaging on melanoma is currently disseminated, most focus on middle-aged NHWs, with a lack of emphasis on material tailored to Hispanic populations [53]. This has led to lower skin cancer awareness among Hispanic individuals, even when comparing Hispanic and non-Hispanic individuals with similar access to healthcare since Hispanic individuals perceived their risk to be lower when compared with non-Hispanic individuals [8].

### 2.4. Language Barriers

Language barriers and preferences significantly impact communication between the patient and healthcare provider, often leading to poorer health outcomes due to the inability to effectively communicate information [30]. For example, Hispanic Spanish-preferring English Medicare patients reported worse experiences than Hispanic English-preferring patients in receiving care in a timely manner [31]. Likewise, Hispanic patients place greater trust in providers who are able to speak Spanish or have Hispanic backgrounds [54]. This can lead to barriers that can also create additional challenges in delivering and receiving appropriate care for the patient.

### 2.5. Cultural Differences and Acculturation

Many studies have recognized provider bias and behaviors as a crucial contributing factor to health inequities, suggesting that cultural and language differences between patients and their healthcare providers may impact their outcomes [55, 56]. Among Hispanics, those with Hispanic physicians were more likely than those with non-Hispanic physicians to be very satisfied with their care, demonstrating that race concordance is a crucial predictor of satisfaction with healthcare quality [57]. Nonetheless, Hispanics are underrepresented in the USA physician workforce, accounting for only 5.7% of USA medical school graduates in 2022-2023 [33].

Cultural values and beliefs held by Hispanic populations also present challenges in effectively serving Hispanic patients. Familism (familismo), a cultural value in which the family is more important than the self, emphasizes family unity, collective well-being, and loyalty, and it may result in underuse of healthcare resources, including home healthcare services [34, 35]. Although there is no consensus on how to measure fatalism (fatalismo), the cultural belief that fate or another external power predetermines events, it has previously been shown to have a small, marginal effect on Hispanic and Latino patient adherence to certain cancer screenings, albeit often being reported among medically underserved patients and patients with limited cancer knowledge [36–38]. Other values and beliefs that are important to consider when delivering healthcare to Hispanic patients include the traditional feminine role (marianismo), traditional masculine role (machismo), folk beliefs, and hot–cold theory of illness beliefs [35, 39].

Acculturation can be defined as the extent to which a person from a nondominant ethnic group assimilates aspects of the dominant ethnic group's culture [58, 59]. The extent to which Hispanic patients are acculturated affects their skin cancer–related behaviors, provider encounters, and adherence. For example, Spanish-acculturated Hispanics had higher rates of preventative skin-cancer behaviors than Hispanics with both high Spanish and high English acculturation, who in turn had higher rates than English-acculturated Hispanics [40]. In Latino populations, higher acculturation levels were associated with increased sunscreen use despite decreased shade and protective clothing usage [41]. Additionally, English-acculturated Hispanics had lower perceived risk of skin cancer and greater perceived suntan benefits [42].

Aside from behavioral effects, low acculturation may also potentially account for the lower trust placed in providers by Hispanics compared with NHW and Black populations [54]. Following this logic, a study determined that lower acculturation correlated with lower cervical cancer screening [43]. However, less acculturated women of Mexican origin reported feeling a lower sense of responsibility and control over their own health and accepted external agents such as chance or doctors as determining their health outcomes. Yet, another study found that although Hispanic women who were less acculturated more strongly believed that healthcare providers control their health, this did not lead to increased adherence [44]. Interestingly, Hispanic patients with a limited health literacy, an indicator of lower levels of acculturation, had higher provider trust than patients with higher health literacy levels [45]. These seemingly contradictory findings indicate a need for more uniform methods of measuring acculturation and additional research.

### 2.6. Prevention and Future Directions

#### 2.6.1. Screening and Early Detection

Early detection of melanoma is crucial as it significantly improves prognosis by identifying the disease at an earlier, more treatable stage [60]. Routine skin checks, self-examination, educational campaigns, and biopsies enable the early detection of melanoma, helping to reduce melanoma mortality rates [61]. Some significant barriers to screening and early detection include lower-income patients who face challenges such as lack of insurance, transportation, and access to specialized care as well as education gaps, resulting in less awareness about the importance of screenings [62].

Some strategies to treat NHW individuals effectively include emerging technologies such as artificial intelligence (AI)-based image analysis, along with sequential digital dermoscopy, teledermatology, or dermatoscopy inspections [63]. Novel AI technologies offer promising opportunities to enhance early melanoma detection and reduce disparities. Multiple instance learning (MIL) algorithms, an AI approach combined with digital image analysis, have shown considerable success in classifying skin lesions [64, 65]. Notably, the DC-SMIL algorithm employs a MIL framework with spherical separation to accurately identify melanoma, dysplastic nevi, and common nevi from dermoscopic images [66]. These AI tools can improve diagnostic accuracy in ambiguous cases, making them valuable for expanding early detection capabilities. Further integrating these AI technologies into teledermatology services could improve access for underserved Hispanic communities by overcoming barriers such as provider shortages and the need for in-person specialist visits. However, it is important to acknowledge that most existing dermatology AI models lack explicit validation across diverse populations and are often limited by dataset size or diversity. Given the differences in skin lesion and melanoma presentation among Hispanic and other skin-of-color populations, future efforts focusing on presentation inclusivity are needed to ensure identification accuracy across all populations [67].

In parallel, broader public health strategies such as community outreach programs, mobile clinics, free screening days, and expanded Medicare coverage can also help address socioeconomic barriers and promote earlier melanoma detection in Hispanic communities.

Culturally tailored health campaigns that take place within the local community such as at schools, churches, or community centers can resonate with the diverse populations [68]. Through these community outreach programs, educational sessions can be held within the community utilizing trusted community leaders to share the importance of screenings while also including information about resources for low-cost or free screenings [69]. Studies have shown that community outreach can significantly increase public awareness and knowledge about diseases and reduce health disparities [70]. By implementing these strategies for melanoma, it can reduce long-term health costs while also increasing preventive care.

Mobile screening units which are traveling clinics that provide cancer screening services outside of traditional clinical settings can also increase early detection and screenings [71]. Mobile mammography units have been successfully implemented in areas where patients face barriers to annual screenings and have been able to provide access to breast cancer detection, significantly increasing screening rates in underserved populations that face barriers to cancer screening [72]. Mobile screening units are equipped with the necessary instruments to bring healthcare services directly to underserved areas, eliminating many barriers including transportation, clinic availability, and specialist care [73]. These mobile screening units can be utilized for skin screenings, improving access to care, enhancing community engagement, and raising awareness about melanoma.

Moreover, free screening days in clinics, hospitals, or pop-up centers can encourage patients who avoid preventive screenings due to cost to access these services. The integration of free health screening days can advance public health efforts allowing for collaboration with the community such as at schools or community organizations, providing essential health services but also creating an opportunity to educate individuals who participate in the screenings [74]. Some examples of screening initiatives across the United States include Cancer Screen Week, which is an annual initiative dedicated to increasing awareness and access to cancer screenings, and National Breast and Cervical Cancer Early Detection Program, which offers free or low-cost mammograms and pap tests to eligible patients focusing on those who are uninsured or underinsured [75]. These free screening have also been implemented into melanoma and include the American Academy of Dermatology Free Skin Cancer Screenings which connects patients with volunteer physicians to conduct the skin screenings [76] and The Sun Bus operated by the Colorado Melanoma Foundation which is a mobile clinic that travels around the United States offering free skin exams and sun-safety education [77]. With the success of these existing programs, they have saved many lives through early detection. Early detection can significantly reduce healthcare costs by avoiding the treatments necessary during late-stage cancer and expanding free melanoma screenings ensures more coverage to patients, so they are able to receive the preventive services they need.

Furthermore, Medicaid has undergone significant changes, specifically with the Affordable Care Act which has expanded its coverage for cancer screenings, making preventive services accessible to more individuals. Currently, 10 states have not adopted the Medicaid expansion. Texas and Florida are notable examples since combined, they account for a significant proportion of the Hispanic population in the United States and have higher rates of uninsured rates of Hispanics when compared with Medicaid expansion states [78]. In Hispanic and Latino populations, Medicaid coverage is crucial because despite them being more likely to take part in the workforce than non-Latinos, they are less likely to be provided by insurance coverage that is sponsored by their employer [79]. By expanding coverage in the nonexpansion states, access to early screening and essential healthcare services for Hispanic and Latino populations could be improved, addressing existing disparities in insurance coverage and ultimately enhancing healthcare outcomes.

#### 2.6.2. Public Health Campaigns

Targeted education may be an effective strategy for mitigating skin cancer disparities in the Hispanic population in the United States. Greene et al. reported increased melanoma knowledge in Latino patients after implementing a targeted digital educational program. Their program involved emailing a random sample of Latino patients, Instagram and Facebook advertisements, and posts on Mayo Clinic's social media channels and websites. In their study, they reported that study participants showed a 22% increase in their melanoma knowledge score and sustained an 11% increase at the 3-month follow-up visit [25]. In a trial online educational melanoma program, Roman et al. provided a 5-min online video about melanoma and then sent a second survey after 1 month. In this trial, a significantly higher proportion of participants answered correctly regarding melanoma prevention and risk factors when compared with the first survey that was given prior to watching the video [26].

Additionally, qualitative explorations aimed at designing culturally relevant tools for skin cancer prevention among Hispanic individuals have suggested social media apps such as WhatsApp would be a promising tool for disseminating melanoma educational materials [27]. Reasons for these particular exploration directions include that a large number of Hispanics are social media users and that WhatsApp is one of the applications most widely used by Hispanics [28]. A potential effective strategy could be to disseminate short videos on platforms such as WhatsApp aimed at promoting melanoma prevention in Hispanic communities. Furthermore, due to the large emphasis on familism, it was also proposed to focus sun-safety practices and campaigns on women within Hispanic families to yield cascading results [27].

Besides digital educational programs, community-based programs have also been proven to be effective. In a pilot program that collaborated with community centers to enhance skin cancer awareness and screening among Latinos in Chicago, most participants improved from “not at all” confident regarding performing skin examinations to “somewhat confident” by attending structured educational sessions led by Spanish-speaking Lay Health Workers and practicing examination techniques with guidance [29]. Overall, both digital and community-based educational programs have shown considerable potential in addressing skin cancer disparities in outcomes within Hispanic populations in the United States. A comprehensive, integrated strategy that combines digital and community-focused efforts in a culturally competent way may be promising in advancing melanoma awareness, prevention, and early detection in Hispanic communities by providing tailored campaign efforts.

#### 2.6.3. Enhancing Medical Education Frameworks

The curricula for medical schools, residency programs, and fellowships should integrate health systems, cultural competence, and health financing topics. Even though this is standard across many medical schools in the United States, there is a need to further tailor these materials to Hispanics in the United States. Medical Spanish programs in healthcare provider training programs may also help address language barriers, with longitudinal Spanish programs being associated with improved Spanish-listening comprehension in medical programs [32]. Further focusing on these topics would better equip physicians to understand and identify financial toxicity, and the specific barriers that Hispanic patients face, such as language, cultural differences, discordant care, and limited access to healthcare resources. Additionally, it would empower Hispanic patients to better understand melanoma-related risks, prevention strategies, encourage screening, and thereby allow for earlier treatment options.

#### 2.6.4. Provider Training

The recent reversal of affirmative action policies may further exacerbate the discordant care experienced by Hispanic patients due to the decreased number of physicians from the underrepresented minorities entering the US healthcare workforce [57]. To alleviate this strain, enhancing provider cultural competence regarding Hispanic cultural values and acculturation could potentially help improve the quality of healthcare for underserved and minority populations. Additionally, the provider's role in reducing patients' financial burden is another topic of consideration.

An important Hispanic cultural value to take into account when delivering healthcare is familism because of its role in decreasing home healthcare services usage. As such, provider management strategies should not only demonstrate acknowledgment of and respect for familism but also strive to encourage optimization of adherence and self-care when deemed appropriate [35]. Interdisciplinary interventions that support these traditional values but simultaneously incorporate skilled support into the home may help to reduce disparities associated with familism [34].

Likewise, acculturation and recognition of ethnic heterogeneity along with the associated language barriers must also be addressed. A potential direction could include the healthcare system usage of Medicaid funding and Health Resources and Services Administration grants to collaborate with local agencies working with settling legal immigrants. Recruited legal immigrants could help create English-language instruction that incorporates healthcare topics for new immigrants [54]. Recruiting higher numbers of qualified Hispanic health providers or incorporating in-person professional interpreters may also result in higher satisfaction and better communication outcomes for patients [54, 80]. Tailoring interventions based on patients' acculturation levels, such as prioritizing knowledge dissemination for Spanish-acculturated Hispanic patients as opposed to educating English-acculturated patients on skin cancer risks, may help to further counteract acculturation disparities [7].

## 3. Related Works

Several prior articles have documented melanoma disparities affecting Hispanic populations. Multiple epidemiological analyses have shown that Hispanics are more likely to receive a late-stage melanoma diagnosis and suffer worse survival outcomes compared with NHWs [53, 81, 82]. Other works have identified key socioeconomic and healthcare barriers, including limited insurance coverage and restricted access to specialty care, contributing to delayed detection and treatment in these communities [53, 83]. Cultural and linguistic barriers, such as language discordance and lower health literacy, have also been previously examined as factors influencing healthcare utilization and outcomes [84].

While valuable, most existing studies tend to focus on isolated determinants such as epidemiology, insurance status, or cultural factors individually [53, 81]. In contrast, this review article provides a comprehensive synthesis by integrating biomedical, socioeconomic, cultural, and technological dimensions influencing melanoma disparities. A novel feature of this work includes its focus on emerging technologies such as AI-based diagnostic tools and evaluation of culturally tailored public health campaigns. Additionally, this review stresses the importance of enhancing medical education frameworks and provider training to improve care quality and trust in Hispanic populations. By bridging these multiple aspects, this article offers a holistic perspective and actionable recommendations to address melanoma disparities in this rapidly growing and underserved population.

## 4. Conclusion

Delayed diagnosis and inferior outcomes in Hispanic melanoma patients are the consequences of pervasive, multifactorial disparities accumulated across healthcare accessibility, socioeconomic, and cultural domains. Addressing these burdens through integration of preventative screenings, targeted educational campaign expansions and culturally sensitive, comprehensive provider training are essential to refining the current healthcare framework in order to deliver improved melanoma treatment to Hispanic patients.

Moving forward, further studies clarifying the etiology of and causal relationships between the risk factors in this population are necessary so as to uncover previously overlooked patterns and ultimately advance equitable patient care. Efforts should also prioritize the adoption of rigorously validated technologies, such as AI-assisted diagnostic tools, to ensure accurate melanoma detection across diverse Hispanic subgroups. Expanding Medicaid coverage and reducing immigration-related barriers remain critical steps to improving access to care. Furthermore, culturally tailored, bilingual educational initiatives delivered through popular social media platforms alongside community outreach can enhance awareness and promote early detection. Strengthening medical education on Hispanic health needs and increasing Hispanic representation in the healthcare workforce will further improve patient–provider communication and foster trust, leading to better outcomes. Ongoing research into the diverse experiences within Hispanic populations is essential for the development of precise, effective interventions that can ultimately reduce melanoma disparities and support long-term health equity in this underserved community.

## Figures and Tables

**Table 1:** Summary of barriers, impacts, and proposed solutions to melanoma disparities in Hispanic populations.

Barriers	Impact	Proposed solutions
Low health insurance among noncitizen Latino immigrants [13]	High uninsured rates leading to decreased access and increased preventable hospitalizations; limited use of specialty care, despite outreach programs [14–16]	Medicaid expansion; community education to reduce immigration-related enrolment fears; mobile clinics and public–private partnerships
Non-Medicaid expansion in certain states (Texas, Florida) [17]	Sustained higher uninsured rates among Hispanics in those states, contributing to poorer access	Advocacy for Medicaid expansion
Socioeconomic challenges (poverty, economic opportunity, low education)	Occupational UV exposure and chemical hazards; poor sunscreen use and low cancer knowledge among outdoor Hispanic workers [18–20]; widening disparities due to low education; nonsignificant increases in melanoma mortality among least educated [21–24]	Targeted skin cancer education emphasizing prevention behaviors; campaigns focused on outdoor workers
Limited health literacy and melanoma awareness	Poor awareness of melanoma risk; lower perception of risk among Hispanics	Tailored digital and community health education, including use of social media and lay health workers [25–29]
Linguistic barriers	Poor communication; delayed diagnosis [30]; Hispanic Spanish-preferring Medicare patients report worse care experiences [31]	Culturally and linguistically tailored education; bilingual providers; medical Spanish training for providers [32]
Provider shortages and underrepresentation of Hispanics in the medical workforce	Lower patient satisfaction linked to race concordance; only 5.7% of U.S. medical graduates are Hispanic, contributing to care discordance [33]	Increase Hispanic medical student recruitment and retention; cultural competence training for providers
Cultural differences, heterogeneity in Hispanic subgroups (nativity, migration) and acculturation	Underuse of healthcare services due to familism (family-centered values); delayed melanoma diagnosis; variable adherence due to fatalism, marianismo, machismo, folk beliefs [34–39]; lower acculturation linked to lower preventive behaviors and variable trust [40–45]	Community- and family-focused culturally competent campaigns; provider training on Hispanic cultural values and acculturation; customized education and intervention programs that address subgroup-specific needs and acculturation level
Insufficient medical education on Hispanic health needs	Providers less aware of cultural, linguistic, and financial barriers faced by Hispanics, impacting care quality	Incorporate Hispanic health and cultural competence training in medical education; medical Spanish programs [32]

## Data Availability

Data sharing is not applicable to this article as no datasets were generated or analyzed during the current study.

## Nomenclature

AI: Artificial intelligence
MIL: Multiple instance learning
NHW: Non-Hispanic white
SES: Socioeconomic status
UV: Ultraviolet
